# REPercussions: how geminiviruses recruit host factors for replication

**DOI:** 10.3389/fmicb.2023.1224221

**Published:** 2023-09-20

**Authors:** Sara Shakir, Muhammad Mubin, Nazia Nahid, Saad Serfraz, Muhammad Amir Qureshi, Taek-Kyun Lee, Iram Liaqat, Sukchan Lee, Muhammad Shah Nawaz-ul-Rehman

**Affiliations:** ^1^Plant Genetics Lab, Gembloux Agro-Bio Tech, University of Liѐge, Gembloux, Belgium; ^2^Virology Lab, Center for Agricultural Biochemistry and Biotechnology, University of Agriculture, Faisalabad, Faisalabad, Pakistan; ^3^Department of Bioinformatics and Biotechnology, Government College University, Faisalabad, Pakistan; ^4^Department of Integrative Biotechnology, Sungkyunkwan University, Suwon, Republic of Korea; ^5^Risk Assessment Research Center, Korea Institute of Ocean Science and Technology, Geoje, Republic of Korea; ^6^Microbiology Lab, Department of Zoology, Government College University, Lahore, Pakistan

**Keywords:** geminiviruses, replication, pathogenicity, virus interaction, host factors

## Abstract

Circular single-stranded DNA viruses of the family *Geminiviridae* encode replication-associated protein (Rep), which is a multifunctional protein involved in virus DNA replication, transcription of virus genes, and suppression of host defense responses. Geminivirus genomes are replicated through the interaction between virus Rep and several host proteins. The Rep also interacts with itself and the virus replication enhancer protein (REn), which is another essential component of the geminivirus replicase complex that interacts with host DNA polymerases α and δ. Recent studies revealed the structural and functional complexities of geminivirus Rep, which is believed to have evolved from plasmids containing a signature domain (HUH) for single-stranded DNA binding with nuclease activity. The Rep coding sequence encompasses the entire coding sequence for AC4, which is intricately embedded within it, and performs several overlapping functions like Rep, supporting virus infection. This review investigated the structural and functional diversity of the geminivirus Rep.

## Introduction

Single-stranded DNA (ssDNA) viruses infect a wide variety of living organisms. According to a recent metagenomic sequencing analysis, these intracellular obligate parasites are widespread and have a diverse host range (Krupovic et al., 2020). Following the molecular structure and phylogeny of replication-associated protein (Rep), the ssDNA viruses have been categorized as circular Rep-encoding single-stranded (CRESS) DNA viruses (phylum: *Cressdnaviricota*). Certain CRESS DNA viruses of eukaryotic origin encode only Rep and coat protein genes. Due to the HUH (two histidine residues separated by hydrophobic residues) endonuclease domain in the Rep, most DNA viruses replicate using a rolling circle mechanism (Chandler et al., 2013). Like other CRESS DNA viruses, members of the family *Geminiviridae* replicate via rolling circle replication (RCR). In addition, the geminivirus Rep (also called AC1, C1, or AL1 in the literature) interacts with the host DNA replication factors for virus DNA replication. The origin of the geminivirus Rep coding sequence is predicted from phytoplasma plasmids using the metagenomic sequence analysis (Krupovic et al., 2009; Kazlauskas et al., 2019). However, the possibility that this shared domain organization in both Rep coding sequences is either the result of convergent evolution or reflects a more recent common ancestry cannot be ruled out at this point. Besides Rep, the geminivirus replication-enhancer protein (REn or AC3) also plays an ancillary role in virus replication.

Genome duplication during the cell cycle is highly regulated in all eukaryotes and is fundamental to preserving genome integrity (Alabert and Groth, 2012; MacAlpine and Almouzni, 2013). This duplication process is known as DNA replication, which aids an organism in transmitting its genetic information to the daughter cells produced during cell division to maintain continuity between generations. DNA replication occurs in three steps: initiation, wherein pairs of replication forks assemble at the replication origin; elongation, wherein forks copy the chromosomes by semiconservative DNA synthesis; and termination, wherein converging replication forks meet (Balakrishnan and Bambara, 2013; Costa et al., 2013). From bacterial to eukaryotic cells, the DNA replication process is highly organized and follows a strict rule of only once per cell cycle (Siddiqui et al., 2013; Skarstad and Katayama, 2013). Geminiviruses largely depend on the host cell machinery and metabolism for replication, transcription, translation, and assembly processes. They manipulate the cells to progress from the resting phase (G0 or G1) to the S phase to create a favorable environment for their replication. This ability allows tropism for cells that otherwise might not be able to support virus DNA replication (De Beeck and Caillet-Fauquet, 1997; Reed et al., 2004). Several host factors have been identified that physically interact with geminivirus Rep. This manuscript reviews the progress in determining the molecular structures and host factors that interact with geminivirus Rep.

## Diverse genomes of geminiviruses

Geminiviruses, with more than 520 species, are recognized by the International Committee on Taxonomy of Viruses (ICTV) as one of the most numerous and diverse groups of plant viruses, causing a wide range of diseases in economically important plants (Zerbini et al., 2017). Naturally, geminiviruses are transmitted by different hemipterous insects. They have been classified into fourteen genera: *Becurto-*, *Begomo-*, *Capula-*, *Citloda-, Curto-*, *Eragro-*, *Grablo-, Maldo-*, *Mastre-*, *Mulcrile-, Ogun-, Topile-, Topocu-*, and *Turncurto-virus*, based on the genome organization, host range, insect vector type, and phylogeny (King et al., 2018; Roumagnac et al., 2022). Members of the genus *Begomovirus* have genomes that are either monopartite, with a single genomic component, DNA-A, of ∼2.6–3.0 kilobases (kb), or bipartite, with two ∼2.6 kb genomic components, DNA-A and DNA-B, together making up the genomic size of around ∼5.2 kb. DNA-A and DNA-B components are packed into twinned quasi-icosahedral virions (Zerbini et al., 2017). Members of other genera comprise the monopartite genome (Zerbini et al., 2017). Geminiviruses have variable genomic structures depending on the genus type. Generally, the DNA-A component encodes four proteins, Rep, REn, transcription activator protein (TrAP/AC2), and pathogenesis/symptom determinant protein (C4/AC4) in the complementary-sense orientation, and two proteins, coat protein (CP/AV1) and pre-coat protein (AV2) in the virion-sense orientation (Devendran et al., 2022). In addition, the geminivirus genomes have been recently identified to encode several small proteins, which have predicted roles in the sub-cellular location and pathogenicity of associated viruses (Gong et al., 2021; Zhao et al., 2022). The DNA-B component encodes movement protein (MP/BC1) in the complementary-sense orientation and nuclear shuttle protein (NSP/BV1) in the virion-sense orientation (Zerbini et al., 2017). Both DNA-A and DNA-B components share a common region (CR) of 180–200 nucleotides that usually spans most of the intergenic region (IR). The IR is a non-coding region encoded by all geminiviruses, which contains cis-acting regulatory elements for gene expression, a predicted hairpin structure containing the conserved (among most geminiviruses) nonanucleotide sequence (TAATATTAC) as part of the loop, and small repeated sequences, known as “iterons,” which are sequence-specific binding sites for Rep. Together, the iterons and hairpin form the origin of replication (ori).

Begomoviruses in the Old World (OW) and a few mastreviruses have been associated with ∼1.4 kb-sized ssDNA molecules referred to as alphasatellites and betasatellites (Zhou, 2013; Kumar et al., 2014; Hamza et al., 2018) and recently assigned to families, *Alphasatellitidae* and *Tolecusatellitidae*, respectively (Adams et al., 2017). Alphasatellites contain an ori similar to other members of the family *Nanoviridae* and encode a nanovirus-like Rep (280–315 amino acids), which enables them to self-replicate. However, they require helper virus components for inter- and intracellular movement and, in exchange, provide helper viruses with selective advantages by acting as suppressors of gene silencing (Nawaz-ul-Rehman et al., 2010). Alternatively, betasatellites do not code for any replication gene and completely depend on the helper component, DNA-A, for their replication, encapsidation, and vector transmission. However, they encode a betaC1 (βC1) protein, which is a suppressor of gene silencing and serves as a major pathogenicity determinant (Zhou, 2013).

**Figure 1:**
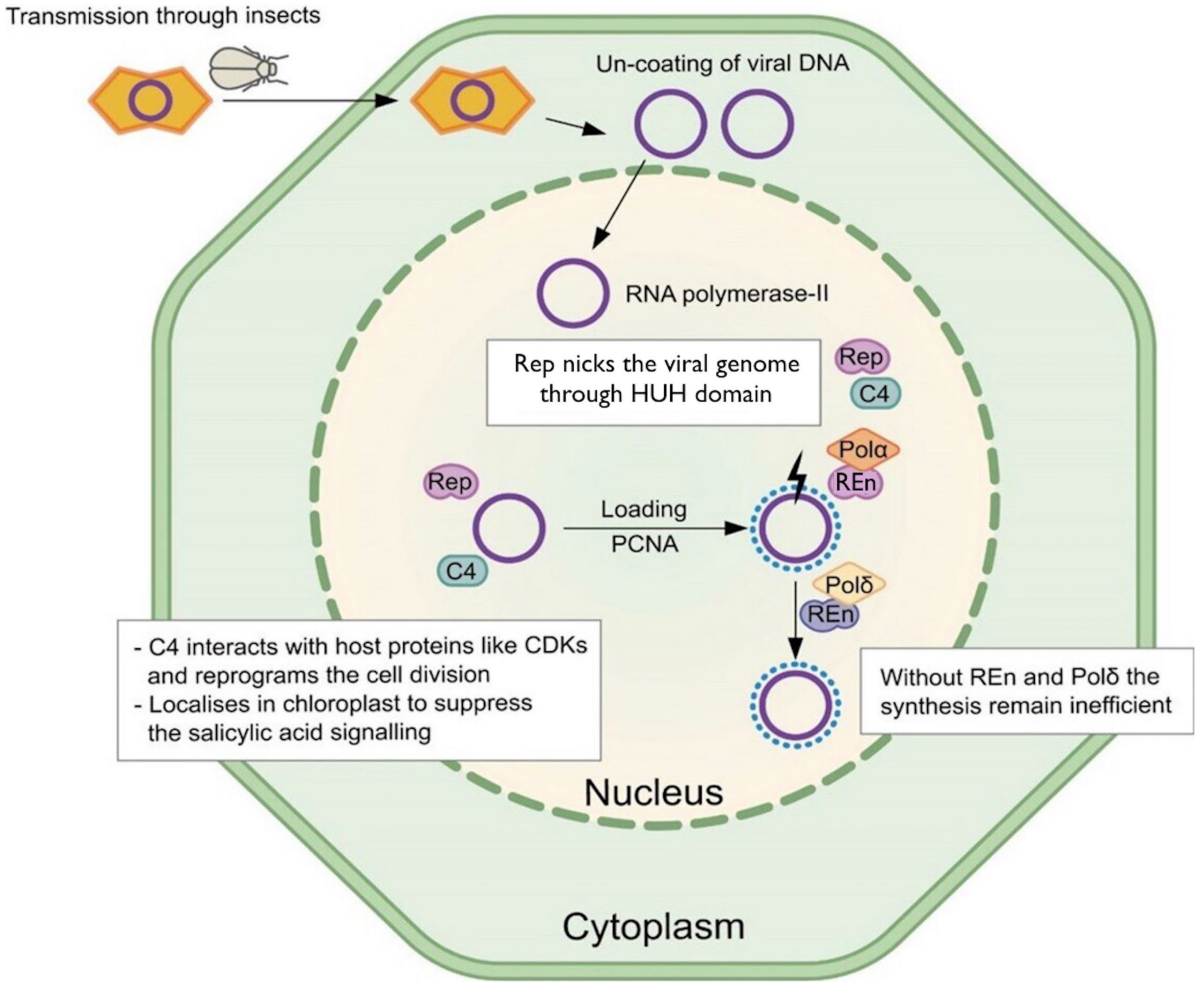
Model for Rep functions. The Rep recruits PCNA to interact with DNA. It binds with nonanucleotide through the HUH domain and starts the rolling circle replication. The C4 protein coded by polycistronic Rep is multifunctional and targets the salicylic acid-based defense pathway. Without REn and Polδ interaction, the replication remains inefficient.

Geminivirus infection occurs in host-differentiated cells, where the virus reprograms the host cell cycle to create a permissive environment for its genome replication (Gutierrez, 2000). Geminiviruses use only two proteins for virus DNA replication, Rep and REn. These virus proteins interact with each other and various host factors, directing the host cell to form replication complexes required for virus DNA replication (Figure 1; Fontes et al., 1994; Gutierrez, 2000). Geminivirus Rep directs virus-specific recognition of its cognate ori at the virus genome and starts virus DNA replication (Fontes et al., 1994). Additionally, Rep regulates its own transcription and the expression of virion-sense genes in some geminiviruses (Hofer et al., 1992). Initial experiments demonstrated that Rep is essential for virus DNA replication, where *Tomato golden mosaic virus* (TGMV) Rep overexpression in *Nicotiana benthamiana* plants supported efficient virus replication mutated for its Rep (Hanley-Bowdoin et al., 1990). Further experiments demonstrated interesting applications of Rep, where overexpression of the mutated version of *Bean golden mosaic virus* (BGMV) Rep resulted in partial resistance in bean plants to the respective viruses (Faria et al., 2006).

## Rep proteins vary at the species level but share similar structures

**Figure 2:**
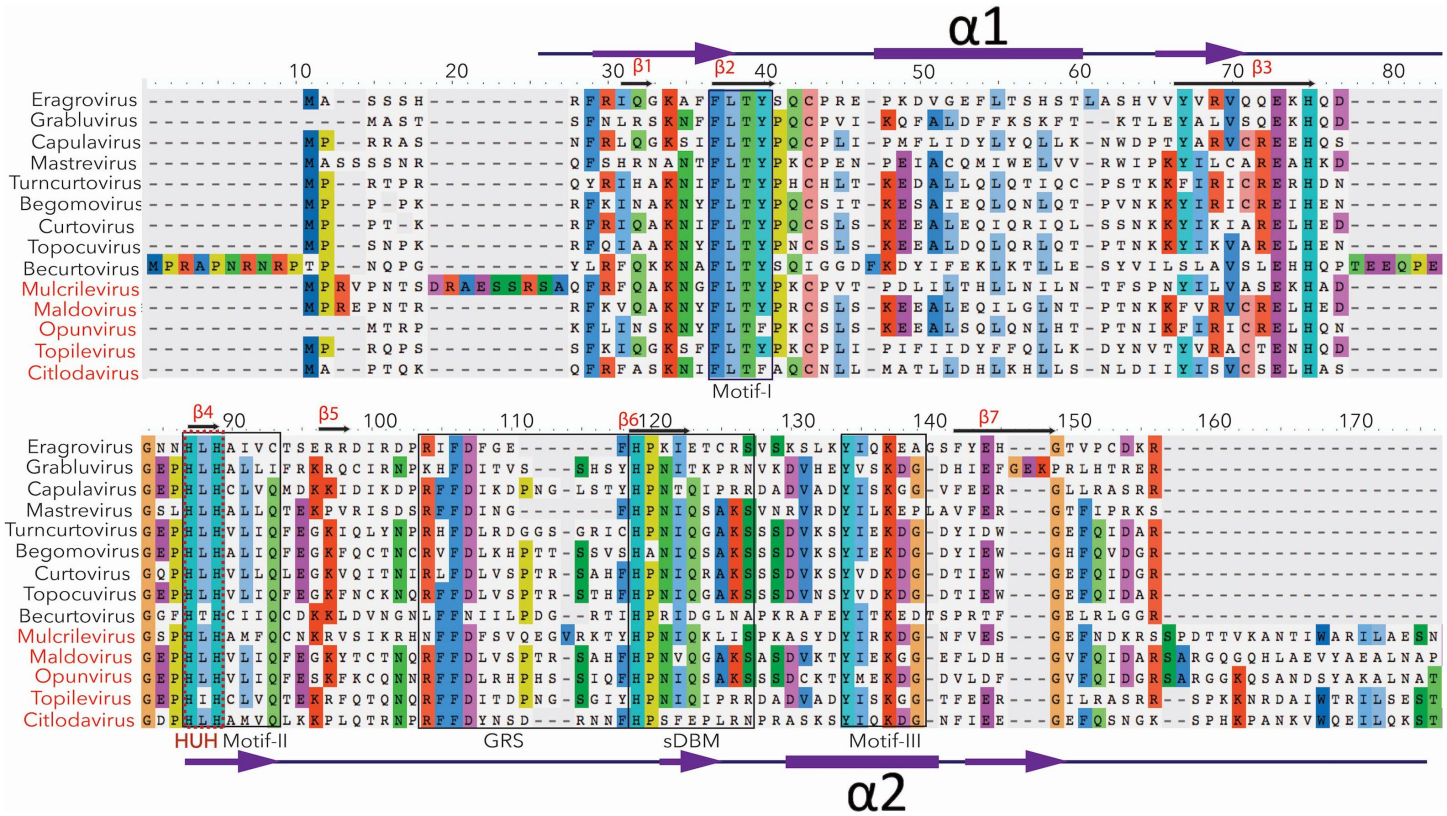
The conserved sequence motifs for Rep of fourteen recognized genera of geminiviruses. The most conserved amino acids are mentioned in different colors. For example, motifs 1, 2, and 3 are conserved among all the geminiviruses. The critical HUH domain of CRESS DNA viruses is mentioned in the red text. HUH domain is involved in rolling circle amplification. The newly recognized five genera (mentioned with red color) have relatively (20%) larger Rep. The conserved part of their Rep is presented here.

**Figure 3:**
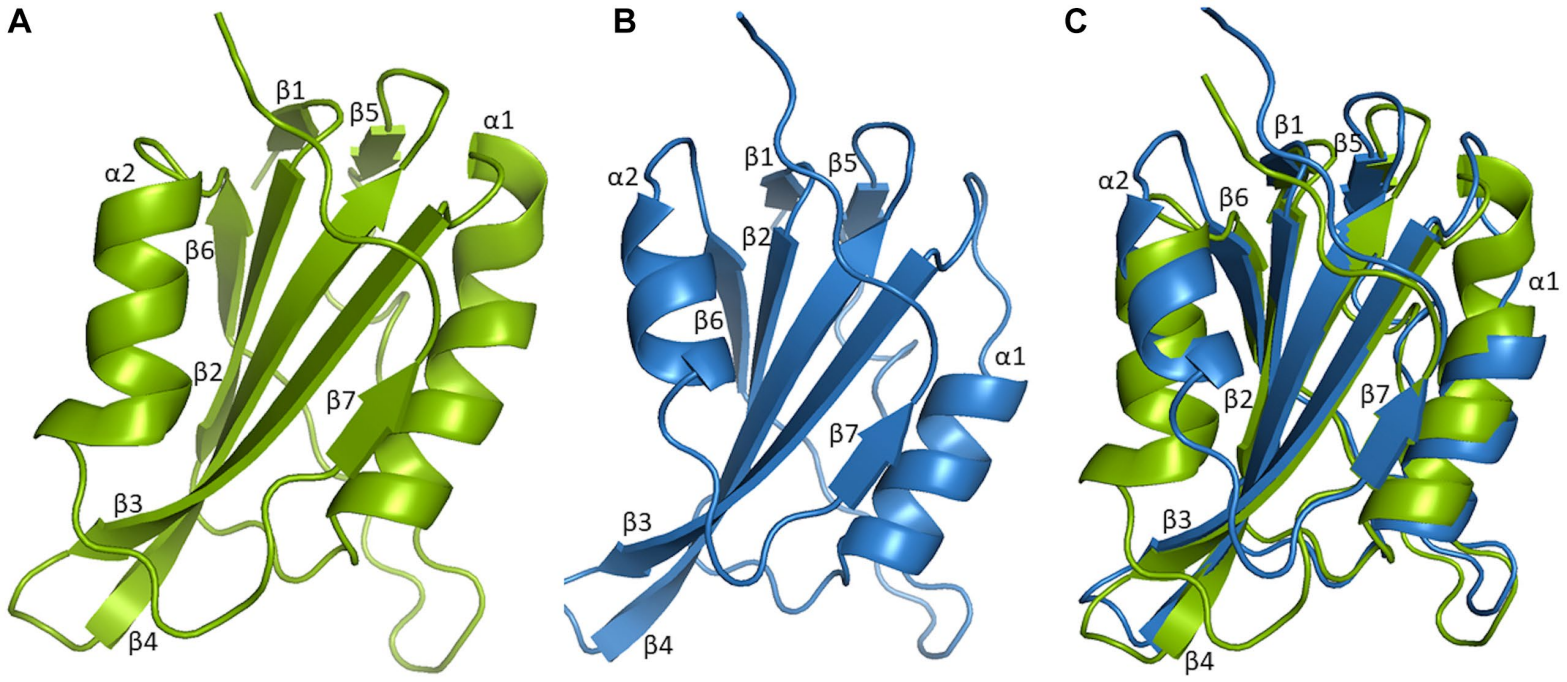
Three-dimensional structural comparison of N-terminal of *Mastrevirus* and *Begomovirus* Rep. Protein models were build using Swiss-model software and visualized in Pymol for Rep of *Mastrevirus, Wheat dwarf virus* (WDV) **(A)**, *Begomovirus, Tomato yellow leaf curl virus* (TYLCV) **(B)**. Superimposition of WDV Rep and TYLCV Rep structure **(C)** indicates highly conserved structures between different two genera, comprised of five antiparallel β-sheets, β2, β3, β4, β6, β7 in the center, flanked by two β-sheets, β1, β5 and two α-helices, α1 and α2 are shown. However, two α-helices are smaller in TYLCV Rep compared to WDV Rep.

Geminivirus Rep is the most conserved protein in sequence, position, and function (Figure 2), while its structure and folding patterns present slight variability among different genera (Figure 3 and Supplementary Figure S1; explained below). It shares domain resemblance with the replication protein of a eubacterial plasmid, implying a strong evolutionary link between these proteins (Ilyina and Koonin, 1992; Koonin and Ilyina, 1992). This is consistent with the idea of geminivirus evolution from prokaryotic ssDNA plasmids (Oshima et al., 2001; Krupovic et al., 2009). Rep, with a size of approximately 360 amino acids, transcribes from a bidirectional core promoter located in the IR except for *Mastre-*, *Capula*, *Becurto*, and *Grablo-virus* Rep (C1:C2), which is a spliced version of *C1* and *C2* open reading frames (ORFs) (Hanley-Bowdoin et al., 1999, 2013; Varsani et al., 2017). In addition to Rep, *Mastre-, Capula-*, *Becurto*-, and *Grablo-virus* also code for replication-associated protein A (RepA/C1) with sizes varying from 260 to 300 amino acids, transcribed from ORF *C1* (Hofer et al., 1992; Collin et al., 1996; Gutierrez et al., 2004; Varsani et al., 2017).

The N-terminal of geminivirus Rep comprises three domains: DNA binding (amino acids, 1–130), DNA cleavage and ligation (amino acids, 1–120), and oligomerization (amino acids, 120–180) domains. It also contains the binding site for various host proteins, such as proliferating cell nuclear antigen (PCNA), retinoblastoma-related (RBR) protein, SUMO (Small Ubiquitin-like Modifiers)-conjugating enzymes (SCE), Geminivirus Rep-interacting kinase (GRIK), and adenosine triphosphatase (ATPase), as well as geminivirus protein REn. The DNA-binding and cleavage/ligation domains contain three motifs (I, II, and III), which are involved in DNA binding, metal binding, and catalyzing endonuclease activity, respectively (Koonin and Ilyina, 1992). Furthermore, it comprises two alpha-helices (helix αI and αII) and a geminivirus conserved motif designated as the geminivirus Rep sequence (GRS), required for replication initiation (Figure 2; Orozco and Hanley-Bowdoin, 1998; Nash et al., 2011).

The C-terminal contains the ATPase domain, including Walker A and Walker B, which are required for the activities of ATPase, helicase, and DNA unwinding process during replication (Desbiez et al., 1995; Orozco et al., 1997; Choudhury et al., 2006; Clérot and Bernardi, 2006; George et al., 2014). Additionally, another relatively conserved cyclin interaction motif (RXL) has been discovered at the C-terminal close to the Walker A motif among all geminivirus genera (except *Becurtovirus*) and has been demonstrated to be indispensable for geminivirus genome replication as a mutation of the RXL motif failed to replicate the virus genome in *N*. *benthamiana* and fission yeast (Nash et al., 2011; Hipp et al., 2014).

So far, the crystal structure of the *Wheat dwarf virus* (WDV) Rep HUH endonuclease domain and the NMR structure of the catalytic domain of *Tomato yellow leaf curl virus* (TYLCV) Rep have been solved (Campos-Olivas et al., 2002; Everett et al., 2019). A three-dimensional structural comparison of the catalytic domain of TYLCV and WDV Rep indicated that it consists of five-stranded antiparallel β-sheets (β2, β3, β4, β6, and β7) in the center, flanked by two-stranded β-sheets (β1 and β5), a β-hairpin, and two α-helices (Figure 3). However, two α-helices appeared more compact in begomoviruses compared to mastreviruses. Intriguingly, Rep of most of the geminivirus genera shared a relatively conserved structure and folding patterns; however, three genera, including *Eragro-*, *Topile*-, and *Capula-virus*, lack two-stranded β-sheets (β1 and β5) in the N-terminal (Supplementary Figure S1). The HUH endonuclease domain directs a metal ion to enable the Rep nicking activity of a specific ssDNA sequence. Therefore, solving its structure provides vital insights into the Rep recognition of ssDNA and sequence specificity (Everett et al., 2019).

Sequence analysis of *Chili leaf curl virus* (ChiLCV) Rep predicted the presence of a nuclear localization signal between the amino acid residues 244 and 273 (Kushwaha et al., 2017). However, a recent study has reported an auxiliary role of lysine residues at the N-terminal (between amino acid residues, 40–108) of Rep for its nuclear localization, and these lysine residues are highly conserved among various geminiviruses. Mutational analysis of the TYLCV Rep single lysine residue (K67A) significantly increased Rep nuclear exclusion, which was further increased when mutations K71A or K101A alone or in combination were added to K67A (Maio et al., 2019).

The *Mastrevirus* RepA is structurally similar in its N-terminal region to Rep, containing DNA binding, cleavage/ligation, and oligomerization domains. However, the functions of these RepA domains have not been experimentally described. The C-terminal of RepA includes the promoter transactivation domain (TRA) and binding sites for RBR and GRAB (geminivirus RepA binding) transcription factors (Horváth et al., 1998; Liu et al., 1999; Xie et al., 1999; Gutierrez et al., 2004; Hefferon et al., 2006). Mutational studies in *Maize streak virus* (MSV) RepA have shown that it is necessary for efficient virus DNA replication and is involved in ssDNA production (Ruschhaupt et al., 2013).

## AC4 coding sequence lies within Rep coding sequence

The coding region for AC4 in both monopartite and bipartite geminiviruses is generally embedded within the Rep (Fondong, 2019) and is absolutely required for virus infection (Hipp et al., 2014; Carluccio et al., 2018). However, depending on the geminivirus species, transgenic plants expressing AC4 display distinct phenotypes (Mills-Lujan and Deom, 2010; Luna et al., 2012). According to the unique target signals, such as a chloroplast transit peptide (cTP) and acylation sites, AC4 has been discovered to interact with membranes in the chloroplast, plasma membrane, nucleus, and cytoplasm, which could account for these functional distinctions among geminivirus AC4 proteins (Luna and Lozano-Durán, 2020). For instance, the AC4 of apple geminivirus is a symptom determinant and is targeted to the nucleus, plasma membrane, and chloroplast (Zhan et al., 2018).

The geminivirus AC4 protein has multiple overlapping functions, just like Rep. One of the functional characteristics of AC4 is its prominent role in reprogramming the cellular environment for virus infection. The cell cycle is tightly regulated by the interplay between cyclins, cyclin-dependent kinases (CDKs), and CDK inhibitors (CKIs). Recently, *Tomato leaf curl Yunnan virus* (TLCYnV) C4 was found to interact with and sequester the phosphorylation ability of glycogen synthase kinase 3 (GSK3) in *N. benthamiana*, a SHAGGY-like kinase and an inhibitor of cyclin CycD1;1, designated as SKη. This interaction leads to the accumulation of CycD1;1, which then forms a complex with CDK and induces cell division (S phase) that enables virus DNA replication (Mei et al., 2018). *Beet severe curly top virus* (BSCTV)-encoded C4-mediated upregulation of RING finger E3 ligase (RKP) in *Arabidopsis* plants is yet another strategy to induce cell cycle progression by *Curtovirus* (Lai et al., 2009). Furthermore, RKP interacts with and downregulates the activity of the cell cycle inhibitors, and upregulates E2F activity, thereby inducing cell proliferation by stimulating cell entry from G1 to S (Cheng et al., 2013).

The AC4 protein of many geminiviruses suppresses the RNA silencing pathway in plants. For example, the AC4 of *Mungbean yellow mosaic virus* (MYMV) targets BARLEY ANY MERISTEM 1 (BAM1), a receptor-like kinase that is a positive regulator of cell-to-cell movement of RNAi signals (Carluccio et al., 2018). Likewise, TYLCV C4 interacts with both BAM1 and BAM2, thus regulating the intercellular spread of RNA silencing signals (Rosas-Diaz et al., 2018). In addition, the AC4 protein of the *Cotton leaf curl Multan virus* (CLCuMuV) targets S-adenosyl methionine synthetase activity, a critical enzyme of the host methyl cycle, thus suppressing both transcriptional and post-transcriptional gene silencing (Ismayil et al., 2018). Similarly, *Tomato leaf curl New Delhi virus* (ToLCNDV) C4 protein interacts with the host AGO2 protein and influences RNA silencing and cytosine methylation of the virus genome (Vinutha et al., 2018). Furthermore, *Tomato leaf curl Palampur virus* (ToLCPalV) AC4 has also been described as a suppressor of gene silencing (Kulshreshtha et al., 2019).

Owing to the co-existence of two subcellular targeting signals, an N-myristoylation site and cTP, the geminivirus AC4 has also been implicated in suppressing chloroplast-mediated plant defense mechanisms. For instance, TYLCV-C4 is transported from the plasma membrane to the chloroplast upon activation of plant defense and suppresses salicylic acid-mediated defense communication between the nucleus and the chloroplast (Medina-Puche et al., 2020). Other functions have also been reported for geminivirus AC4; for instance, TYLCV C4 has been implicated in virus transport inside phloem tissues (Rojas et al., 2001).

## Rep interacts with virus/satellite DNA and initiates replication

The geminivirus Rep starts RCR by locating and attaching to the CR, which contains the iteron sequences between the TATA box and the transcription start site (Fontes et al., 1992). Rep recognizes the virus-specific ori, and this recognition mechanism depends on the architecture of the ori, which appeared to be different between *Begomovirus* and *Mastrevirus* (Gutierrez et al., 2004). The ori in *Mastrevirus* consists of a large cis-acting region with several Rep-binding sites, whereas the ori in *Begomovirus* has a single Rep-binding site (Fontes et al., 1992; Sanz-Burgos and Gutiérrez, 1998; Castellano et al., 1999). However, various begomovirus species have different iteron sequences and ways of attaching Rep to these repetitive regions. For instance, the iteron sequence in the TGMV consists of 13 bp sections with two 5 bp repeats. *In vitro* binding analysis showed that Rep binding to these iteron sequences is necessary to initiate virus DNA replication (Fontes et al., 1994). However, *Tomato leaf curl virus* (TLCV) Rep-binding regions consist of direct repeat “GGTGTCT” elements in CR, as demonstrated by footprinting analysis (Akbar Behjatnia et al., 1998). In addition, *Mungbean yellow mosaic Indian virus* (MYMIV) contains an additional iteron sequence downstream of CR (Singh et al., 2008). In the case of mastreviruses, Rep binds to three sites, located upstream and downstream of the ori and at the base of the stem-loop structure (Laufs et al., 1995; Castellano et al., 1999).

Once Rep binds to the ori using its binding motif I, it cleaves the phosphodiester bond between the seventh and eighth residues of the invariant loop structure 5’TAATATTAC3′ that involves its motif II (Heyraud-Nitschke et al., 1995; Laufs et al., 1995; Orozco and Hanley-Bowdoin, 1998; Arguello-Astorga et al., 2004). Another sequence in motif III is also involved in cleavage and binding activity (Orozco et al., 1997), thus providing a free 3’OH end to start the synthesis of nascent virus DNA by host polymerases. The elongation involves extending this 3′ end using a complementary strand with the help of host polymerases and associated factors. This is evident from Rep interaction with various host factors, such as replication protein A 32 (RPA32), PCNA, replication factor C (RFC), and minichromosome maintenance 2 (MCM2) (Luque et al., 2002; Bagewadi et al., 2004; Singh et al., 2007). Rep recognizes and cleaves the nonanucleotide in newly formed viral circular DNA and lures its 3’ end to a previously synthesized 5′ end using its DNA-binding domain located in the N-terminus, resulting in the formation of a new viral genome-sized unit (Laufs et al., 1995). In addition to motifs I–III, binding and cleavage activities by two α-helices located between motifs I and II are necessary for virus DNA replication (Orozco and Hanley-Bowdoin, 1998). Moreover, GRS is required for Rep binding. The artificial zinc-finger protein-based mutation of GRS in TYLCV Rep lost its ability to cleave and initiate virus DNA replication (Nash et al., 2011). Many other cis-elements, such as TATA and G boxes, CA and AG motifs, and spacing between the Rep-binding site and hairpin, have also been demonstrated to control virus DNA replication (Orozco et al., 1997). The oligomerization domain located in the center of the Rep is crucial for Rep oligomerization and ultimately for virus DNA replication, as TGMV mutants for its oligomerization domain are defective in replication (Orozco et al., 1997). Rep oligomerization has also been illustrated for mastreviruses, MSV and WDV (Horváth et al., 1998; Sanz-Burgos and Gutiérrez, 1998; Castellano et al., 1999; Missich et al., 2000). In the case of WDV, both Rep and RepA form large oligomeric nucleoprotein C and V complexes by binding near the TATA box of complementary and virion sense promoters, respectively. However, DNase1 footprinting analysis demonstrated that their binding regions were not identical. In plants, bimolecular fluorescence complementation (BiFC) assay in *N. benthamiana* demonstrated *Abutilon mosaic virus* (AbMV) Rep self-interaction to form oligomers and their accumulation in the nucleoplasm (Krenz et al., 2011).

Besides Rep, REn actively contributes to virus DNA replication. It recruits host DNA polymerase alpha (α) and delta (δ) to the replicase complex and also interacts with virus Rep (Settlage et al., 1996, 2005; Wu et al., 2021). *In vitro* studies with the *Tomato leaf curl Kerala virus* (ToLCKeV) have shown that REn enhances the ATPase activity of Rep (Pasumarthy et al., 2010). REn also modulates the cellular environment by interacting with host RBR and NAC domain-containing transcription factors (Selth et al., 2005; Settlage et al., 2005). The ability of REn to oligomerize may also play a role in virus DNA replication, as evidenced by interacting TGMV and BGMV Rep and REn, which allowed the formation of heteromultimeric units and virus DNA replication in tobacco protoplasts (Settlage et al., 1996). Geminivirus CP might also regulate the virus DNA replication as its strong interaction with Rep has been illustrated for MYMIV, which results in the downregulation of Rep nicking and ligation ability (Malik et al., 2005).

The Rep protein allows replication of its cognate DNA-B component by binding to specific cis-elements in the CR (Lazarowitz et al., 1992). However, the synthesis of viable pseudo-recombinants between TGMV DNA-A and *Tomato yellow spot virus* (ToYSV) DNA-B, which do not have identical Rep-binding regions, demonstrates that trans-replication of only its cognate DNA-B molecule is not a strict rule (Andrade et al., 2006).

Monopartite begomoviruses are often associated with betasatellites, which are necessary to induce distinctive disease symptoms. However, betasatellites depend on the helper virus for replication, movement, and encapsidation of their genomes. Betasatellite DNA can be replicated by distinct geminiviruses, but they prefer cognate over non-cognate helper viruses (Qing and Zhou, 2009). For example, *Cotton leaf curl Multan betasatellite* (CLCuMuB) and *Ageratum yellow vein betasatellite* (AYVB) can be trans-replicated using CLCuMuV, *Ageratum yellow vein virus* (AYVV), and *Eupatorium yellow vein virus* (EpYVV), but not by *Honeysuckle yellow vein virus* (HYVV) (Saunders et al., 2008). It has been reported that *Tobacco curly shoot betasatellite* (TbCSB) and *Tomato yellow leaf curl China betasatellite* (TYLCNB) can be trans-replicated by their non-cognate helper viruses, but when co-inoculated, cognate betasatellites predominated over non-cognate partners in the later stages of infection (Qing and Zhou, 2009). These studies found that, while trans-replication of betasatellites occurs from non-cognate helper viruses, there is some specificity for trans-replication of heterologous satellite DNA.

Similar to Rep binding sequences in the virus ori, different sequence motifs have been identified in betasatellites for the Rep binding of the helper virus. For example, a G motif at 143 bp upstream of βC1 has been identified in CLCuMuB, which is important for betasatellite DNA replication (Eini et al., 2009). The sequences within the 26–295 nt region were essential for Rep binding and TLCV satellite DNA replication (Li et al., 2007). The involvement of the satellite conserved region (SCR) region in the DNA replication of AYVB, TYLCCNB, and TbCSB has been experimentally indicated (Saunders et al., 2008; Zhang et al., 2016). DNase I footprinting analysis revealed that betasatellites contain homologous iteron-like sequences of approximately 260 nt known as Rep-binding motifs (RBM) for specific recognition by Rep of their cognate helper viruses. This RBM is located immediately upstream of the satellite-conserved rolling circle cruciform structure and has a higher Rep-binding affinity for cognate helper viruses than non-cognate helper viruses (Zhang et al., 2016). The deletion of the RBM motif demonstrated its important role in efficient betasatellite DNA replication. However, more recently, cis-elements have been characterized for TbCSB for specific Rep binding by its cognate helper virus, *Tobacco curly shoot virus* (TbCSV) (Xu et al., 2019). Competitive DNA-binding assays revealed that TbCSB contains two iteron-like repeat elements (5′-GGACC-3′), which are identical to repeat sequences in TbCSV that are responsible for specific Rep binding; when these sequences were mutated, TbCSB lost its ability to prefer cognate helper virus-mediated replication (Xu et al., 2019). Sequence analysis has revealed that, interestingly, species-specific repeats in betasatellites are homologous to the repeats in cognate helper viruses. In addition to specific repeats, general repeats (5′-GGTAAAT-3′) have been identified upstream of SCR that allow Rep binding by non-cognate helper viruses.

## Rep as RNAi suppressor and pathogenicity protein

Plants use cytosine and histone methylation as a defense against invading geminiviruses, thus regulating virus DNA replication and transcription of virus genes (Raja et al., 2010). *Arabidopsis* plants mutated for methylation such as cytosine methyltransferases (CMT) and histone methyltransferases (MET) supported high levels of geminivirus DNA replication, and virus DNA isolated from such plants was less methylated (Raja et al., 2008). *In vitro* methylation of geminivirus DNA results in reduced virus DNA replication in tobacco protoplasts, primarily due to the downregulation of virus gene transcription (Brough et al., 1992; Ermark et al., 1993). Another study found that geminiviruses’ heterogeneous linear double-stranded (ds) DNA form was preferentially methylated among different replicative intermediates, namely, open circular (oc), covalently closed circular (ccc), and heterogeneous linear DNA (Paprotka et al., 2011). However, to counter-defense the host response, geminiviruses encode several suppressors of host-mediated transcriptional gene silencing (TGS) and post-transcriptional gene silencing (PTGS) (Rishishwar and Dasgupta, 2019). Geminivirus Rep-mediated modification of the plant epigenome and downregulation of host methyl cycle enzymes, MET, and CMT, have been demonstrated in *N. benthamiana* (Rodríguez‐Negrete et al., 2013). In the case of mastreviruses, both Rep and RepA of WDV suppress the spread of RNA silencing signals, thus inducing both local and systemic silencing suppression of the *GFP* gene (Liu et al., 2014; Wang et al., 2014). Gel mobility shift assays showed that the Rep of WDV binds to 21 and 24 nt small interfering RNA (siRNA) duplexes and single-stranded (ss)-siRNA interfering host mechanism of PTGS. Moreover, deletion mutagenesis of the Rep showed that both N- and C-terminals are not required for silencing suppression, while the N-terminal is required for pathogenicity (Wang et al., 2014).

In addition to geminivirus Rep, alphasatellite-encoded Rep also induced PTGS suppression. The Rep from various alphasatellites, such as Gossypium darwinii symptomless alphasatellites (GDarSLA), Gossypium mustelinium symptomless alphasatellite (GMusSLA), and Cotton leaf curl Multan alphasatellites (CLCuMuA), are known PTGS suppressors (Nawaz-ul-Rehman et al., 2010; Abbas et al., 2019). However, the exact mechanisms of RNAi suppression remain largely unknown.

## Rep as a transcription regulator

Rep has been shown to regulate the transcription of early and late-expressing virus genes (Eagle et al., 1994; Shung and Sunter, 2007). Rep binds to the iteron sequences at CR and represses the activity of its own promoter, which leads to the expression of TrAP and REn as the transcription start site of these genes lies within the coding region of Rep. However, replication initiation and transcriptional autoregulation are unrelated, as Rep mutants that are unable to initiate replication could still regulate the transcription of virus genes (Eagle et al., 1994). Mutational analysis identified an RGG motif between 124 and 126 amino acids in the Rep of *Tomato yellow leaf curl Sardinia virus* (TYLCSV) responsible for its transcription autoregulation activity (Sardo et al., 2011). Furthermore, geminivirus Rep-mediated recruitment of host machinery involved in post-translational modification at virus chromatin has been associated with the activation of virus gene transcription (explained below) (Lee et al., 2007; Kushwaha et al., 2017).

## Global geminivirus Rep–host proteins interaction studies

Upon geminivirus infection, Rep is the first virus protein synthesized and is responsible for reprogramming the host cell cycle for virus DNA replication and transcription of other virus genes. Therefore, it interacts with many host proteins implicated in replication, post-translational modification, and defense machinery (Table 1). RBR proteins are the key factors controlling cell cycle progression in both plants and animals. A similar role in controlling virus DNA replication has been described in wheat cells, where virus DNA accumulation is significantly reduced upon expression of the RBR protein (Xie et al., 1996). Both Rep and RepA of WDV interact with plant RBR proteins through their LXCXE motif. This interaction is important for geminivirus DNA replication as its disruption abolishes virus DNA replication in wheat cells (Xie et al., 1995). However, contrary to WDV RepA, the other geminivirus Rep does not contain the LXCXE motif; instead, it binds to the RBR protein through other motifs. For example, TGMV Rep interacts with RBR protein via its novel helix 4 motif, which is located between Rep’s 101 and 180 amino acid regions. Rep’s helix 4 motif is made up of a hydrophobic core flanked by charged amino acid residues, and these residues play an important role in Rep-RBR binding. In addition to Rep, REn interacts with the RBR protein through polar residues at the N- and C-terminals (Settlage et al., 2001, 2005).

**Table 1:** Host factors interacting with geminivirus Rep, their function in the plant cell, and virus infection.

Viral protein	Host protein name and function	Type of interaction with Rep and result	References
WDV-RepA WDV-Rep TGMV-Rep	Plant retinoblastoma-related protein (RBR) involves cell cycle regulation and differentiation	Positive interaction (PI): reprograms the cell cycle to the S phase and is essential for virus DNA replication	Xie et al. (1995), Collin et al. (1996), and Kong et al. (2000)
TYLCSV-Rep MYMIV-Rep	Proliferating cell nuclear antigen (PCNA), involved in DNA replication and repair	Negative interaction (NI): downregulates Rep helicase and ATPase activity, inhibits RCR, and controls viral DNA copy number	Castillo et al. (2003) and Bagewadi et al. (2004)
WDV-Rep	Replication factor C (RFC) acts as clamp loader of PCNA for DNA polymerase	Assembles the elongation complex for virus DNA replication	Luque et al. (2002)
MYMIV-Rep	Replication protein A-32 (RPA32): required for DNA replication, repair, recombination, and metabolism	Downregulate and upregulate helicase and ATPase activity of Rep, driving replication from initiation to elongation phase	Singh et al. (2007)
MYMIV-Rep	Minichromosome maintenance protein 2 (MCM2) involves in the initiation and regulation of DNA replication	Essential for virus DNA replication. The exact function is unknown	Suyal et al. (2013b)
TGMV-Rep CaLCuV -Rep	Histone (H3) participates in transcription initiation and elongation via chromatin remodeling	Assist in virus DNA replication and transcription via displacing nucleosome barriers on viral minichromosomes	(Kong and Hanley-Bowdoin (2002))
TGMV-Rep CaLCuV-Rep	Geminivirus Rep-interacting kinase (GRIK) involves spindle fiber alignment, regulates gene transcription, etc.	Inhibits cell entry into the mitotic phase	Kong and Hanley-Bowdoin (2002)
TGMV-Rep TYLCSV-Rep ACMV-Rep	SUMO-conjugating enzyme (SCE1), a nuclear protein involves in the conjugation pathway, controls mitosis, and S and M phase cyclin degradation	Rep-SCE1 interferes with PCNA sumoylation, thus creating a favorable environment for virus infection	Castillo et al. (2004) and Sánchez-Durán et al. (2011)
MYMIV-Rep	RAD51 involves DNA recombinational repair	Promotes virus recombination-dependent DNA replication	Suyal et al. (2013b)
MYMIV-Rep	RAD54: involves DNA recombinational repair	Promotes virus recombination-dependent DNA replication	Kaliappan et al. (2012)
WDV RepA	Geminivirus RepA binding protein (GRAB1 and GRAB2), involved in plant growth, development, and senescence	NI: inhibits virus DNA replication in plants	Xie et al. (1999)
MYMIV-Rep	NAC083, member of NAC domain-containing protein family, a multifunctional protein involved in ribosome biogenesis, stress response, etc.	Required for virus DNA replication	Suyal et al. (2014)
ChiLCV-Rep	Histone ubiquitination 1 (HUB1) involves in ubiquitination of histone 2B protein, regulating plant development, and stress response.	Rep recruits post-translational machinery on the viral promoter, ubiquitinate histone 2B protein, and activates transcription of virus genes	Kushwaha et al. (2017)
ChiLCV-Rep	Ubiquitin-conjugating enzyme 2 (UBC2), involved in ubiquitination of host proteins, regulating the expression of genes involved in plant growth and development	Rep recruits chromatin-modifying enzymes on viral minichromosomes, trimethylated histone 3 protein, and activates transcription of virus genes	Kushwaha et al. (2017) and Wu et al. (2021)
TYLCV Rep and REn	The plant DNA polymerases α and δ are required for leading and lagging strands of DNA replication. Geminivirus Rep interacting EWS-like protein 1 (GRIEP-1)	Physically interact with Rep and REn to synthesize geminivirus DNA copies. Positively interacts with Rep protein, however the direct role in virus DNA replication is unknown.	Maio et al. (2020) and Wu et al. (2021)

Geminivirus infection induces the expression of PCNA, as evident from its high accumulation in the infected mature cells (Nagar et al., 2002). Both Rep and REn of TYLCSV and *Indian mung bean yellow mosaic virus* (IMYMV) interact with tomato, tobacco, and pea PCNA, respectively (Schalk et al., 1989; Castillo et al., 2003). Additionally, Rep of OW geminiviruses, including *Pedilanthus leaf curl virus* (PeLCV) and CLCuMuV, has been shown to interact with not only plant PCNA but also yeast PCNA, suggesting a high conservation of Rep binding domains in PCNA among eukaryotic species (Shakir et al., 2021). The middle domain of TYLCV-REn is comprised of hydrophobic residues, and the 132–180 amino acid stretch of TYLCV Rep has been described as crucial for interactions with PCNA (Bagewadi et al., 2004; Settlage et al., 2005). PCNA inhibits the site-specific nicking and ATPase activities of Rep. Since RCR of viruses largely depends on Rep helicase and ATPase activities, it has been proposed that PCNA might act as an inhibitor of RCR, controlling the virus DNA copy number in the infected plant cells (Bagewadi et al., 2004).

The WDV Rep binds to and recruits the larger subunit of replication factor C (RFC) to the 3′ end of nicked geminivirus DNA, assisting in the assembly of the virus DNA replication elongation complex in wheat cells (Luque et al., 2002). *In vitro* studies have demonstrated that the geminivirus Rep interacts with replication protein A (RPA), a single-stranded (ss) DNA-binding protein primarily involved in replication, repair, and recombination (Singh et al., 2007). The 32 kDa subunit of RPA (RPA32) interacts with the C-terminal of MYMIV-Rep and regulates its helicase and ATPase activities, respectively. Therefore, it has been speculated that this interaction might inhibit RCR initiation and play an important role in DNA strand elongation. *In planta*, experiments confirmed that RPA32 positively influences MYMIV DNA replication (Singh et al., 2007). Furthermore, MYMIV-Rep interacts with the minichromosome maintenance 2 (MCM2) subunit, which is the licensing factor of the DNA replication machinery. Yeast and *Arabidopsis* plant mutants for MCM2 displayed reduced virus DNA replication. However, the exact role of MCM2 in viral DNA replication remains unclear (Suyal et al., 2013a).

The assembly of virus genomic DNA as minichromosomes has been previously described for AbMV (Pilartz and Jeske, 2003). *Cabbage leaf curl virus* (CaLCuV) and TGMV Rep interact with the histone-3 (H3) protein of *Arabidopsis*. This interaction might help displace histones from the virus nucleosomes, thus helping in virus DNA replication and transcription processes (Kong and Hanley-Bowdoin, 2002). Moreover, ChiLCV Rep interacts with host proteins involved in post-translational modification, *N. benthamiana*-encoded histone ubiquitination-1 (NbHUB1) and ubiquitin-conjugating enzyme 2 (NbUBC2), and recruits these proteins on the promoter sites at virus minichromosomes (Kushwaha et al., 2017). This recruitment aids in the ubiquitination of H2B and methylation of H3 proteins at virus chromatin, which is associated with RNA polymerase II activation and promoter modification, both of which result in transcription activation (Lee et al., 2007; Kushwaha et al., 2017).

Rep also engages in the geminivirus-related Ser/Thr kinase known as GRIK and the kinesin known as the geminivirus Rep interacting motor protein (GRIMP) (Kong and Hanley-Bowdoin, 2002) that attaches to the spindle fibers. Thus, Rep interaction with kinesin might inhibit cell entry into the mitotic phase. Rep-interacting kinases GRIK1 and GRIK2 accumulate to high levels in CaLCuV-infected tobacco leaves. While these kinases share homology with yeast Sucrose Nonfermenting 1 (SNF1) and mammalian AMPK and with SNF1-related kinases (SnRK1) in plants, it has been hypothesized that these kinases activate the SnRK1 signaling cascade, ensuring the availability of prerequisite precursors and energy sources for virus propagation. In exchange, TGMV-encoded Rep binding to the virus genome is impaired by SnRK1-mediated phosphorylation of the binding domain’s Ser-97 residue, which eventually prevents virus DNA replication (Shen et al., 2018).

Geminiviruses are also known to replicate by recombination-dependent replication (RDR) in addition to RCR (Jeske et al., 2001). Rep interacts with the repair/recombination proteins RAD51 and RAD54 to aid RDR of the virus genome (Kaliappan et al., 2012; Suyal et al., 2013b). Higher expression of RAD51 has been reported in plant cells infected with MYMIV (Suyal et al., 2013b). Complementation assays described the role of these proteins in virus DNA replication, where yeast mutants for *rad51* and *rad54* displayed reduced virus DNA replication while it was restored upon expression of RAD51 and RAD54 proteins. The binding domains have been mapped to the N-terminal of RAD54 and the oligomerization domain of MYMIV Rep (Kaliappan et al., 2012). *In vitro* studies have shown that this interaction enhances both the helicase and ATPase activities of Rep. However, the studies on *Arabidopsis* revealed that the recombination mediator protein RAD51D (a paralog of RAD51) increases *Euphorbia yellow mosaic virus* (EYMV) DNA replication (Richter et al., 2015, 2016). This implies the possible role of other recombination proteins in virus DNA replication and functional redundancy among them (Castillo et al., 2004).

The Rep of TYLCSV, TGMV, and *African cassava mosaic virus* (ACMV) interact with sumo-conjugating enzyme 1 (SCE1) to enhance virus DNA replication (Castillo et al., 2004; Sánchez-Durán et al., 2011). One such host protein, PCNA, is involved in virus DNA replication, which controls the cell cycle, DNA replication, and DNA repair. PCNA shifts among these functions through post-translational modifications. Using a reconstituted sumoylation system in *E. coli*, it has been demonstrated that tomato PCNA sumoylation occurs at two residues, K164 and K254, which play a vital role in DNA metabolism. However, geminiviruses’ Rep expression suppresses this PCNA sumoylation, thus creating a favorable environment for virus DNA replication (Arroyo-Mateos et al., 2018). The lysine residues (K68 and K96) at the N-terminus of TGMV Rep were used in mapping studies to pinpoint the location of this interaction. *In vivo* experiments with the mutation in lysine residues of the Rep did not result in successful virus DNA replication (Castillo et al., 2004; Sánchez-Durán et al., 2011). Furthermore, transgenic tobacco plants mutated for SCE1 did not support virus DNA replication, suggesting the importance of these enzymes in virus DNA replication.

Moreover, the C-terminal of WDV RepA interacts with the N-terminal domains of GRAB1 and GRAB2, belonging to the NAC domain-containing protein family, which is involved in plant development, senescence, and stress response (Xie et al., 1995). When GRAB was expressed in cultivated wheat cells, WDV DNA replication was reduced, indicating a negative interaction between RepA and GRAB proteins. Furthermore, MYMIV Rep has been shown to interact with *Arabidopsis* NAC083, another NAC domain-containing protein and host transcription factor (Suyal et al., 2014). However, the precise function of the Rep-NAC083 interaction in the virus infection cycle has not yet been thoroughly examined (Suyal et al., 2014).

Recently, a global interaction network between the TYLCV and *N. benthamiana* proteins has been presented, which provides a comprehensive overview of potential host proteins targeted by TYLCV during infection (Wang et al., 2017). Additionally, the TYLCV Rep also interacts with EWS-like protein-1 (GRIEP1) and the subunit 4A of the THO complex known as ALY1 (Maio et al., 2020). The direct involvement of GRIEP1 and ALY1 in the virus DNA replication remains unclear. However, their cellular function is to control RNA splicing and the transport of mRNA from the nucleus to the cytoplasm. These observations indicate the indirect involvement of certain host factors in the geminivirus infection cycle.

## Conclusion

Geminiviruses reshape the host’s intercellular environment for their infection and counter-defense against host resistance. Due to their small genome size and limited coding capacity, geminivirus proteins have evolved to perform multiple functions and interact with several host factors involved in cellular pathways. One such virus protein is Rep, which is expressed early in the virus infection and has evolved to possess multiple domains to perform diverse functions in the host cell. Rep is one of the highly conserved proteins, structurally and functionally, across geminivirus species and genera, which facilitates virus DNA replication through reprogramming the host cell cycle and mediating the initiation, elongation, and termination of virus and associated DNA-B and betasatellite genome replication. It also controls the transcription of other virus genes, including TrAP, REn, and its own. Moreover, it acts as a pathogenicity determinant by functioning as a suppressor of gene silencing, which is one of the basic host anti-viral defense responses. Furthermore, the AC4 coding sequence embedded with the Rep coding sequence plays multiple overlapping functions as Rep, including cell cycle progression, RNA silencing suppression, and the intercellular movement of virus DNA molecules.

## Future directions

From the work of the past many years, it is clear that geminivirus–host interaction is complex and involves diverse cellular pathways, ranging from cell cycle to gene silencing. Rep interaction with several host cellular factors has been described in several geminivirus species, but the exact underlying mechanisms remain to be examined in detail for several of these interactions. The study of geminivirus DNA replication events, a better understanding of the crosstalk between Rep and cellular factors, and the effect of these interactions on cellular pathways will increase our understanding of how viruses manipulate host cellular machinery and how certain pathways contribute to disease. Moreover, a global virus–host interaction network will also be instrumental in increasing our understanding of which interactions are conserved and critical for virus infection and how to disrupt them without disturbing plant growth and development. Finally, developing broad-spectrum resistance to geminivirus infections will enable us to target the most conserved pathways among all geminivirus species.

## Author contributions

SSh and MN-u-R: conceptualization. SSh, MM, IL, and SSe: software and drawing. SSh and NN: review of literature. SSh, SL, and MQ: writing and editing. SSh and MN-u-R: supervision and final draft. All authors have read and agreed to the published version of the manuscript.

## Funding

This study was partly supported by a 2022 fund from the Research of Animal and Plant Quarantine Agency, Korea, and from the Korea Institute of Marine Science & Technology Promotion (KIMST) funded by the Ministry of Oceans and Fisheries, Korea (20210466).

## Conflict of interest

The authors declare that the research was conducted in the absence of any commercial or financial relationships that could be construed as a potential conflict of interest.

## Publisher’s note

All claims expressed in this article are solely those of the authors and do not necessarily represent those of their affiliated organizations, or those of the publisher, the editors and the reviewers. Any product that may be evaluated in this article, or claim that may be made by its manufacturer, is not guaranteed or endorsed by the publisher.

## Supplementary material

SUPPLEMENTARY FIGURE S1 Three-dimensional structural comparison of N-terminal of geminivirus Rep. Protein models were build using Swiss-model software and visualized in Pymol for the representative species of *Mastrevirus* **(a)**, *Begomovirus* **(b)**, *Curtovirus* **(c)**, *Grabulavirus* **(d)**, *Becurtovirus* **(e)**, *Citlodavirus* **(f)**, *Turncurtovirus* **(g)**, *Multrilevirus* **(h)**, *Opunvirus* **(i)**, *Topocovirus* **(j)**, *Maldovirus* **(k)**, overlay of a-k **(l)**, *Eragrovirus* **(m)**, *Topilevirus* **(n)**, *Capulavirus* **(o)**, overlay of m-o **(p)**. Superimposition of 11 different genera **(a-k)** of family geminiviridae indicates highly conserved structures comprised of five anitparallel β-sheets, β2, β3, β4, β6, β7 in the center, flanked by two β-sheets, β1 and β2 and two α-helices, α1 and α2 **(l)**. However, two β-sheets, β1 and β2 are missing in three genera, *Eragrovirus, Topilevirus*, and *Capulavirus* Rep **(p)**.
