# Supplementary figures and images for: REPercussions: how geminiviruses recruit host factors for replication
Source: Front Microbiol. 2023 Sep 20;14:1224221. doi: 10.3389/fmicb.2023.1224221 (PMC10548238; doi:10.3389/fmicb.2023.1224221)

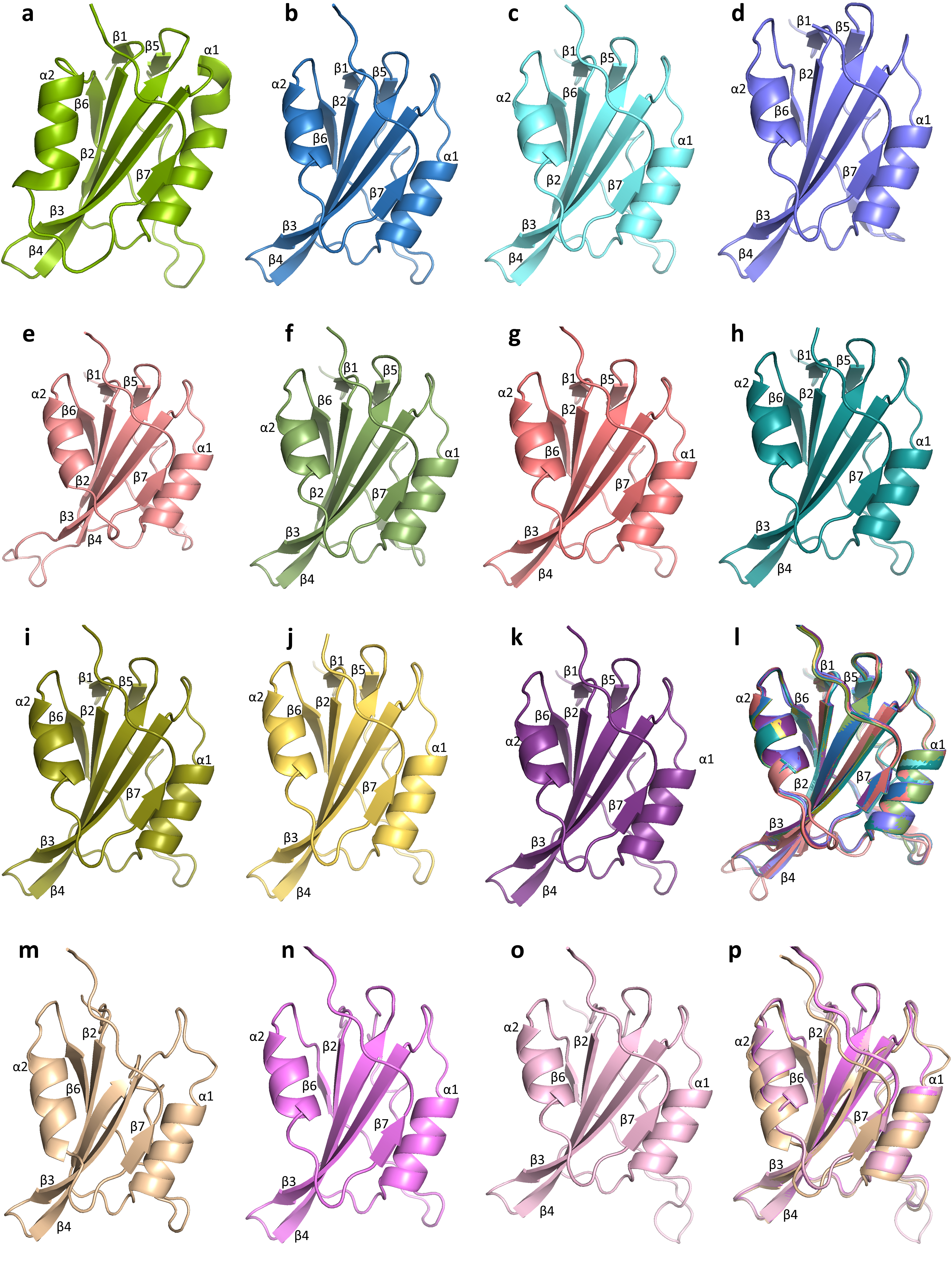

Supplement: SUPPLEMENTARY FIGURE S1 — Three-dimensional structural comparison of N-terminal of geminivirus Rep. Protein models were build using Swiss-model software and visualized in Pymol for the representative species of Mastrevirus (a), Begomovirus (b), Curtovirus (c), Grabulavirus (d), Becurtovirus (e), Citlodavirus (f), Turncurtovirus (g), Multrilevirus (h), Opunvirus (i), Topocovirus (j), Maldovirus (k), overlay of a-k (l), Eragrovirus (m), Topilevirus (n), Capulavirus (o), overlay of m-o (p). Superimposition of 11 different genera (a-k) of family geminiviridae indicates highly conserved structures comprised of five anitparallel β-sheets, β2, β3, β4, β6, β7 in the center, flanked by two β-sheets, β1 and β2 and two α-helices, α1 and α2 (l). However, two β-sheets, β1 and β2 are missing in three genera, Eragrovirus, Topilevirus, and Capulavirus Rep (p). [file Image_1.JPEG]
